# Research on intrinsic capacity as a predictor of falls and disability in community-dwelling elderly

**DOI:** 10.3389/fragi.2025.1589369

**Published:** 2025-05-30

**Authors:** Aihong Liu, Yanjie You, Yumei Wang, Ling Li, Jinrong Yuan

**Affiliations:** Wuhan Union Hospital, Tongji Medical College, Huazhong University of Science and Technology, Wuhan, Hubei, China

**Keywords:** elderly, intrinsic capacity, falls, disability, community elderly care

## Abstract

**Background:**

With aging, a decline in intrinsic capacity can lead to functional impairments, thereby increasing the risk of adverse health outcomes.

**Objectives:**

This study aims to explore the prediction of intrinsic capacity decline on adverse health outcomes, such as falls and disability, over the course of 1 year. By addressing the gap in longitudinal research on community populations in China, the study seeks to deepen the local understanding of healthy aging theory, providing theoretical support for the development of elderly health intervention strategies tailored to Chinese contexts.

**Methods:**

A convenience sampling method was employed to select 248 community-dwelling elderly participants. Over a 1-year follow-up period, the outcomes of falls and disability were monitored. Logistic regression analysis was used to evaluate the relationship between intrinsic capacity and these outcomes.

**Results:**

Among the 248 participants, 46 (19%) experienced falls, and 31 (12.8%) became disabled during the follow-up year. The locomotive dimension (OR = 25.87, 95% CI: 2.95–227.03), psychological dimension (OR = 25.29, 95% CI: 6.45–99.28), and sensory dimensions (OR = 10.75, 95% CI: 2.92–39.56) were identified as significant risk factors for falls. For disability, the locomotive dimension (OR = 4.15, 95% CI: 0.97–17.72), cognitive dimension (OR = 11.27, 95% CI: 3.51–36.18), and psychological dimension (OR = 4.58, 95% CI: 1.69–12.40) were significant risk factors.

**Conclusion:**

Decline in intrinsic capacity serves as an independent predictor of both falls and disability among community-dwelling elderly individuals over the course of 1 year. Early identification of elderly individuals with decreased intrinsic capacity, along with targeted interventions based on different intrinsic capacity levels, can effectively reduce the incidence of falls and disability.

## Introduction

As economic growth accelerates, global population aging is becoming increasingly pronounced. According to the United Nations Department of Economic and Social Affairs in its World Population Prospects 2022, the global population reached 8 billion in 2022, and the proportion of people aged 65 and older is projected to rise from 10% to 16% between 2022 and 2050 (Department of Economic and Social Affairs Population Division, 2022). China is one of the fastest-aging countries in the world. Due to rising life expectancy and declining mortality rates, it is estimated that by 2040, 28% of China’s population will be aged 60 and above (World Health Organization, 2019). The most significant challenge posed by this demographic shift is the complex and severe health issues that the elderly face.

In 2015, the World Health Organization (WHO) introduced the concept of “healthy aging” in its Global Report on Ageing and Health, defining it as the process of developing and maintaining the functional ability necessary for wellbeing in old age (World Health Organization, 2015). Unlike traditional disease-focused approaches, healthy aging emphasizes the positive functional capabilities of older adults, rather than simply the accumulation of health deficits. According to the WHO, functional ability is a comprehensive manifestation of an individual’s intrinsic capacity within a specific environment. Intrinsic capacity (IC) refers to the total combination of physical and mental abilities that an individual can mobilize at any time (World Health Organization, 2015). IC encompasses five dimensions: locomotive, cognitive, psychological, sensory, and vitality, all of which are crucial for predicting health outcomes in the elderly (Beard et al., 2022; Cesari et al., 2018). Unlike functional ability, which may fluctuate due to acute health events, IC emphasizes inherent biological and psychological assets that are independent of external factors (Chhetri et al., 2021). While functional status focuses on current task-specific capabilities, IC reflects a cumulative decline in physiological reserves, which is less reversible. This distinction highlights the predictive value of IC for long-term health outcomes, whereas functional ability and status are more sensitive to short-term interventions.

Intrinsic capacity not only represents the overall health level of the elderly but also serves as a key driver of healthy aging. It can be viewed as an objective measure of the physical and mental well-being of older adults (Dzierżanowski et al., 2020). As individuals age, a decline in intrinsic capacity can lead to functional impairments, increasing the risk of adverse health outcomes such as falls, hospitalizations, disabilities, reduced quality of life, and even death (Tay et al., 2023). International research has shown that, compared to chronic diseases and frailty, intrinsic capacity is a better predictor of falls and disability in community-dwelling elderly individuals (Jia et al., 2023; Prince et al., 2021). Falls are not only a risk factor for disability but also exacerbate the disabilities in elderly individuals, who, due to limited locomotive function, are more prone to subsequent falls (Braun, 1998). Therefore, monitoring and early intervention of intrinsic capacity may help prevent the occurrence of falls and disabilities.

Despite its importance, few longitudinal cohort studies in China have explored the relationship between intrinsic capacity and falls or disabilities. One study involving 196 elderly inpatients found a significant correlation between decreased intrinsic capacity and impaired Activities of Daily Living (ADL) (OR = 1.631, 95% CI: 1.162–2.287) and Instrumental Activities of Daily Living (IADL) (OR = 2.701, 95% CI: 1.736–4.204) (Zhang J. et al., 2020). Another study with 125 elderly inpatients (average age: 81.8 years) found that the more dimensions of intrinsic capacity were impaired, the greater the risk of falls (OR = 2.425, 95% CI: 1.132–4.848) (Zhang D. et al., 2020). However, these studies were cross-sectional and retrospective, and all participants were inpatients. A recent longitudinal study on age-related factors linked falls, functional limitations, and reduced quality of life to intrinsic capacity, emphasizing that falls and disabilities are influenced not only by age but also by individual factors (Meng et al., 2023). This study suggests that intrinsic capacity plays a more specific and clear role in predicting falls and disabilities, particularly in community settings.

The community is the primary living environment for older adults and a key focus of primary healthcare and preventive medicine (Mount et al., 2016). As medical models shift from “treatment-oriented” to “prevention-oriented,” preventing adverse health outcomes in the elderly becomes a crucial strategy for promoting their overall health (Hu et al., 2022). Based on the theoretical framework of healthy aging, this study focuses on community-dwelling elderly individuals as key recipients of primary healthcare services. Through a 1-year follow-up survey, we systematically monitored the dynamic changes in intrinsic capacity among this population and explored its association with falls and disability. By selecting disability and falls as core observational endpoints, this longitudinal research design overcomes the limitations of previous cross-sectional studies and aims to establish temporally sequential evidence chains between intrinsic capacity and adverse health outcomes. The results are expected to achieve two main objectives: firstly, to explore the relationship between the decline in intrinsic capacity and the occurrence of falls and disabilities, providing a foundation for promoting clinical research on intrinsic capacity in China; and secondly, to fill the gap in longitudinal research on community populations in China and deepen the localization of healthy aging theory, offering theoretical support for the development of elderly health intervention strategies with Chinese characteristics.

## Methods

### Design and sample

This study employed a convenience sampling method to survey community-dwelling elderly individuals from two communities in Hannan District, two communities in Hannan District, Wuhan, Zhuankou Street and Junshan Street, between December 2022 and January 2024.

The inclusion criteria for participants were as follows: (1) Community-dwelling elderly individuals aged 60 years or older; (2) Individuals who have lived in the community for more than 3 years; (3) Willingness to participate in the study. The exclusion criteria were: (1) Bedridden elderly individuals; (2) Individuals with hemiplegia or epilepsy; (3) Individuals with mental illnesses; (4) Life expectancy less than 6 months.

Based on the principle that the sample size should be 5–10 times the number of independent variables, and considering 19 variables with an estimated 20% attrition rate, the target sample size was calculated to be between 114 and 228 participants. A total of 248 community-dwelling elderly individuals were enrolled in the study.

### Variables and instruments

#### Demographic and clinical characteristics

This section includes variables such as gender, age, marital status, education level, living situation, BMI, number of illnesses, medication count, smoking history, alcohol consumption, and use of assistive devices.

#### Intrinsic capacity assessment

Intrinsic capacity (IC) was assessed across five dimensions, following WHO guidelines: locomotive, cognitive, psychological, sensory, and vitality. The assessment methods were as follows: Locomotive dimension: Assessed using the Short Physical Performance Battery (SPPB), which includes balance, mobility, and lower limb function, with a total score of 12. A score ≤8 indicates reduced physical capacity (Gomez et al., 2013). Cognitive dimension: Evaluated using the Mini-Mental State Examination (MMSE), with a total score of 30. A score below the following thresholds indicates cognitive decline: ≤19 for those with primary education or below, ≤22 for secondary education, and ≤24 for university education (Katzman et al., 1988). Psychological dimension: Assessed using the 15-item Geriatric Depression Scale (GDS-15), where a score ≥5 indicates poor psychological performance (D’ath et al., 1994). Sensory dimension: Evaluated via self-reported hearing and vision impairment. Vitality: Measured using the Mini Nutritional Assessment Short Form (MNA-SF), with scores ≤11 indicating malnutrition (Rubenstein et al., 2001). Each dimension was scored dichotomously according to the WHO’s Integrated Care for Older People (ICOPE) manual (World Health Organization, 2017): a score of 1 indicates a decline in function, and a score of 0 indicates no decline. Higher scores indicate greater decline in intrinsic capacity.

#### Follow-up on adverse health outcomes

After discharge, a 1-year follow-up was conducted via phone or face-to-face interviews to monitor incidents of disability and falls.

Disability: The Katz Index of Activities of Daily Living (ADL) was used to assess disability (Katz, 1983), with six items: eating, bathing, bowel/bladder control, toileting, dressing, and indoor mobility. If any item was rated as “needs partial help” or “completely dependent,” the individual was considered disabled (Zhang et al., 2023).

Falls: According to the International Classification of Diseases, a fall is defined as a sudden, unintentional change in position, resulting in a person ending up on the ground or a lower surface (WHO Collaborating Center for the Classification of Diseases, Peking Union Medical College Hospital, 1996).

### Data collection

This longitudinal observational study took place from December 2022 to January 2023. Two trained researchers conducted in-person assessments to collect baseline data. Both researchers were qualified in comprehensive geriatric assessment and proficient in evaluation criteria and locomotive function testing methods. Informed consent was obtained from all participants and their families after explaining the study’s purpose, significance, and procedures. Baseline data were collected through face-to-face surveys with standardized instructions, and questionnaires were reviewed for accuracy and completeness. Any missing or incomplete responses were corrected. Follow-up assessments were conducted 1 year later through phone or in-person interviews to record occurrences of falls and disability.

### Statistical analysis

Statistical analysis was performed using IBM SPSS version 23.0. Normally distributed data were presented as mean ± standard deviation (Mean ± SD), and independent sample t-tests were used for group comparisons. Skewed data were expressed as median (interquartile range) [M (P25, P75)], with Mann-Whitney U tests used for group comparisons. Categorical data were presented as percentages or proportions, with chi-square or Fisher’s exact tests applied for group comparisons. Logistic regression models were used to control for confounding factors, selecting variables with statistical significance (P < 0.05) from univariate analysis to identify factors influencing disability and falls. A p-value of less than 0.05 was considered statistically significant.

### Ethics statement

This study received ethical approval from the Medical Ethics Committee of Union Hospital, Tongji Medical College of Huazhong University of Science and Technology, China (Approval No. 0312). The researchers provided a detailed explanation of the study’s objectives to the participants, ensuring their understanding of the research purpose. Participants were assured that all data would be anonymized. Written informed consent was obtained from each participant, who were also given the right to withdraw from the study at any time without any consequence.

## Results

### Characteristics of the participants

A total of 248 participants met the inclusion criteria. Of these, 6 participants (2.4%) were lost to follow-up within the 1-year period due to the inability to contact them or their family members. As a result, 242 participants were included in the final analysis. The median age of the participants was 71 years, and 145 (59.9%) were male. Among the participants, 93.8% were married, and 69.4% were on multiple medications. There were no statistically significant differences observed when comparing the baseline characteristics of the study population (see Table 1).

**TABLE 1:** Baseline characteristics of community-dwelling elderly.

Item		Baseline (non-lost) (n = 242)	Baseline (total) (n = 248)	*P*-Value
Age (IQR)		71 (67, 76)	71 (60, 95)	0.984
BMI (Mean ± SD)		23.69 ± 3.28	23.74 ± 3.28	0.875
Gender				0.957
	Male	145 (59.9)	148 (59.7)	
	Female	97 (40.1)	100 (40.3)	
Marital Status				0.747
	Married	227 (93.8)	231 (93.1)	
	Unmarried or Widowed	15 (6.2)	15 (6.1)	
Education Level, n (%)				0.998
	Primary school or Below	59 (24.4)	60 (24.2)	
	Middle School	83 (34.3)	87 (35.1)	
	High School	71 (29.3)	72 (29.0)	
	University	29 (12.0)	29 (11.7)	
Living Status, n (%)				0.981
	Living Along	49 (20.2)	50 (20.2)	
	Not Living Alone	193 (79.8)	198 (79.8)	
Smoking History, n (%)				0.977
	Yes	29 (12.0)	30 (12.1)	
	No	213 (88.0)	218 (87.9)	
Alcohol Consumption History				0.966
	Yes	28 (11.6)	29 (11.7)	
	No	213 (88.0)	218 (87.9)	
Use of Assistive Devices				0.996
	Yes	42 (17.4)	43 (17.3)	
	No	200 (82.6)	205 (82.7)	
Fall History, n (%)				0.915
	Yes	33 (13.6)	43 (17.3)	
	No	209 (86.4)	215 (86.7)	
Polypharmacy, n (%)				0.987
	Yes	168 (69.4)	172 (69.4)	
	No	74 (30.6)	76 (30.6)	
Number of Diseases (M±SD)		2.09 ± 1.15	2.10 ± 1.14	0.892
Intrinsic Capacity Decline, n (%)		90 (37.0)	93 (37.5)	0.944
	Physical Dimension	83 (34.3)	85 (34.3)	0.996
	Vitality	70 (28.9)	72 (29.0)	0.979
	Cognitive Dimension	54 (22.3)	54 (21.8)	0.885
	Psychological Dimension	39 (16.1)	39 (15.7)	0.906
	Sensory Dimension	45 (18.6)	46 (18.5)	0.989
Falls		46 (19.0)	46 (18.5)	1.000
Disability		31 (12.8)	31 (12.5)	1.000

### Intrinsic capacity levels among community-dwelling elderly

**FIGURE 1:**
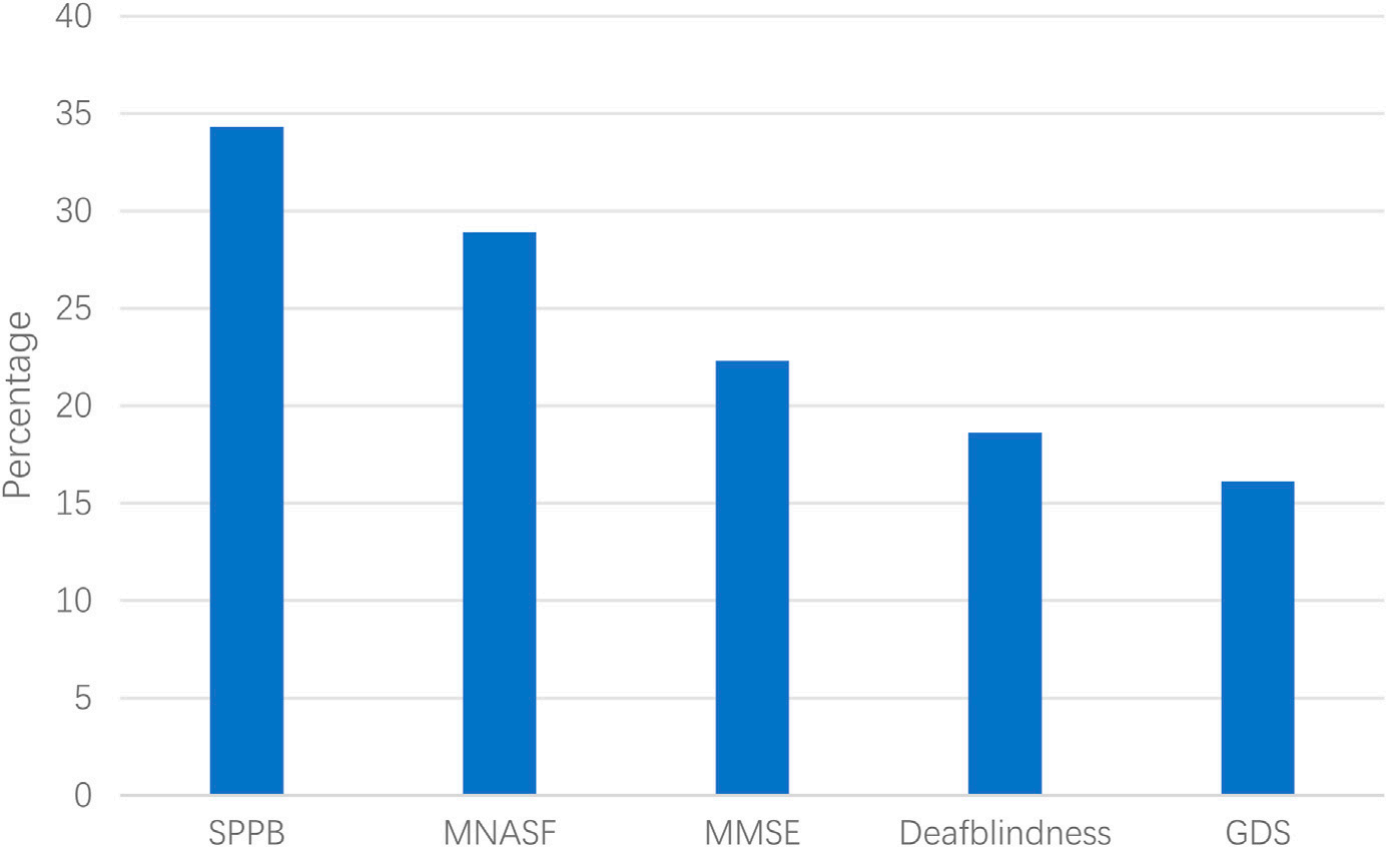
Proportion of intrinsic capacity decline in various dimensions.

Results showed that 90 (37.0%) of the 242 elderly participants exhibited a decline in intrinsic capacity. The greatest decline was observed in the locomotive dimension (34.3%), followed by vitality (28.9%), cognitive dimension (22.3%), sensory dimension (18.6%), and psychological dimension (16.1%) (see Figure 1).

### Comparison of baseline characteristics and intrinsic capacity dimensions between participants with and without disability and falls over one year

Univariate analysis indicated that factors such as age, education level, living alone, use of assistive devices, history of falls, number of illnesses, and all five dimensions of intrinsic capacity were associated with disability. Similarly, age, education level, living alone, use of assistive devices, multiple medication use, history of falls, number of illnesses, and all five dimensions of intrinsic capacity were associated with falls. However, these analyses did not control for demographic confounders such as age and gender. Therefore, multivariate logistic regression analysis was performed to control for these confounders and identify independent factors influencing disability and falls (see Table 2).

**TABLE 2:** Comparison of baseline characteristics and intrinsic capacity dimensions between patients with and without disability and falls during one-year follow-up.

Item		Non-disabled group	Disabled group	*P*-Value	Non-fall group	Fall group	*P*-Value
Baseline characteristics							
Age		69 (65, 73)	77 (68, 82)	<0.001	69 (65, 73)	74 (69, 81)	<0.001
Female		81 (38.4)	16 (51.6)	0.161	77 (39.3)	20 (43.5)	0.602
Marital Status		13 (6.2)	0 (0.0)	0.385	11 (5.7)	2 (4.3)	1.000
Education Level, n (%)				0.046			0.044
	Primary and Below	47 (22.3)	12 (38.7)		43 (21.9)	16 (34.8)	
	Middle School	71 (33.6)	12 (38.7)		65 (33.2)	18 (39.1)	
	High School	64 (30.3)	7 (22.6)		65 (33.2)	6 (13.0)	
	University	29 (13.7)	0 (0.0)		23 (11.7)	6 (13.0)	
Living Alone		163 (77.3)	30 (96.8)	0.012	151 (77.0)	42 (91.3)	0.030
Smoking History		166 (78.7)	25 (80.6)	0.802	153 (78.1)	38 (82.6)	0.496
Alcohol Use		183 (87.1)	30 (96.8)	0.118	169 (86.7)	44 (95.7)	0.087
Assistive Device		189 (89.6)	11 (35.5)	<0.001	176 (89.8)	24 (52.2)	<0.001
Polypharmacy		151 (71.6)	17 (54.8)	0.059	145 (74.0)	23 (50.0)	0.001
Fall History		192 (91.0)	17 (54.8)	<0.001	187 (95.4)	22 (47.8)	<0.001
Number of Disease		1.97 ± 1.04	2.87 ± 1.52	<0.001	1.93 ± 1.02	2.76 ± 31.40	<0.001
BMI		23.81 ± 3.27	22.84 ± 3.29	0.785	23.90 ± 3.24	22.79 ± 3.31	0.57
Intrinsic Capacity Decline							
	Locomotive Dimension	55 (26.1)	28 (90.3)	<0.001	38 (19.4)	45 (97.8)	<0.001
	Vitality	46 (21.8)	24 (77.4)	<0.001	32 (16.3)	38 (82.6)	<0.001
	Cognitive Dimension	28 (13.3)	26 (6.9)	<0.001	26 (13.3)	28 (60.9)	<0.001
	Psychological Dimension	20 (9.5)	19 (61.3)	<0.001	7 (3.6)	32 (69.6)	<0.001
	Sensory Dimension	26 (12.3)	19 (61.3)	<0.001	12 (6.1)	33 (71.7)	<0.001

### Relationship between decline in intrinsic capacity dimensions and disability and falls

Multivariate logistic regression analysis was conducted on statistically significant variables from the univariate analysis, adjusting for confounders such as age, education level, living situation, use of assistive devices, history of falls, and number of illnesses. Results showed that decline in the locomotive, vitality, cognitive, psychological, and sensory dimensions were all independent risk factors for both disability and falls (see Tables 3, 4).

**TABLE 3:** Multivariate logistic regression analysis of the relationship between intrinsic capacity dimensions and disability.

Intrinsic capacity dimension	Disability				
	*β*-Value	SE	Wald	ORa (95%CI)	*P*-value
Locomotive Dimension	2.87	0.74	15.17	17.56 (4.15, 74.27)	<0.001
Vitality	1.63	0.55	8.68	5.11 (1.73, 15.14)	0.003
Cognitive Dimension	3.18	0.62	26.15	24.11 (7.12, 81.63)	<0.001
Psychological Dimension	2.40	0.59	16.46	11.03 (3.46, 35.18)	<0.001
Sensory Dimension	1.62	0.63	6.67	5.06 (1.48, 17.32)	0.01

**TABLE 4:** Multivariate logistic regression analysis of the relationship between intrinsic capacity dimensions and falls.

Intrinsic capacity dimension	Falls				
	*β*-Value	SE	Wald	OR_*a*_ (95%*CI*)	*P*-Value
Locomotive Dimension	5.14	1.12	21.00	171.04 (18.97, 1542.29)	<0.001
Vitality	2.58	0.53	24.18	13.22 (4.72, 36.99)	<0.001
Cognitive Dimension	1.81	0.46	15.33	6.08 (2.46, 15.02)	<0.001
Psychological Dimension	3.88	0.62	39.78	48.54 (14.53, 162.21)	<0.001
Sensory Dimension	3.50	0.65	29.18	33.01 (9.28, 117.41)	<0.001

### Prediction of falls and disability within one year based on decline in intrinsic capacity

A stepwise logistic regression model was constructed to predict falls and disability, incorporating basic characteristics (with P < 0.05 from the univariate analysis) and significant intrinsic capacity decline variables (with P < 0.05 from the adjusted logistic regression model). Results are presented in Tables 5, 6. The locomotive dimension (ORm = 25.87, 95% CI: 2.95–227.03, P = 0.003), psychological dimension (ORm = 25.29, 95% CI: 6.45–99.28, P < 0.001), and sensory dimension (ORm = 10.75, 95% CI: 2.92–39.56, P < 0.001) were predictive factors for falls. The locomotive dimension (ORm = 4.15, 95% CI: 0.97–17.72, P = 0.055), cognitive dimension (ORm = 11.27, 95% CI: 3.51–36.18, P < 0.001), and psychological dimension (ORm = 4.58, 95% CI: 1.69–12.40, P < 0.001) were predictive factors for disability (see Tables 5, 6).

**TABLE 5:** Stepwise logistic regression analysis predicting falls base on intrinsic capacity dimensions.

Intrinsic capacity dimension	Falls				
	*β*-Value	SE	Wald	OR_m_ (95%CI)	*P*-Value
Locomotive Dimension	3.25	1.11	8.62	25.87 (2.95, 227.03)	0.003
Psychological Dimension	3.23	0.70	21.45	25.29 (6.45, 99.28)	<0.001
Sensory Dimension	2.38	0.67	12.77	10.75 (2.92, 39.56)	<0.001

**TABLE 6:** Stepwise logistic regression analysis predicting disability base on intrinsic capacity dimensions.

Intrinsic capacity dimension	Disability				
	*β*-Value	SE	Wald	OR_m_ (95%CI)	*P*-Value
Locomotive Dimension	1.42	0.74	3.69	4.15 (0.97, 17.72)	0.055
Cognitive Dimension	2.42	0.60	16.56	11.27 (3.51, 36.18)	<0.001
Psychological Dimension	1.52	0.51	8.96	4.58 (1.69, 12.40)	<0.001

## Discussion

### Level of intrinsic capacity decline among community-dwelling elderly

This study found that the prevalence of intrinsic capacity decline among community-dwelling elderly individuals was 37.0%. This figure is consistent with the findings of Pan et al. (2024) but lower than the results reported by Lu et al. (2023), which may be attributed to differences in age. The study by Lu et al. involved individuals aged 75 and above, with an average age of 84.0 ± 4.4 years, significantly higher than the average age of 70.2 years in the current study. While intrinsic capacity decline is common in elderly populations, its prevalence varies across studies, ranging from 39.9% to 93.4% (Zhu et al., 2022), likely due to differences in the tools used for assessment. In this study, the most significant declines were observed in the locomotive, vitality, and cognitive dimensions, which aligns with findings by (Ma et al., 2021). Other recent studies have reported sensory impairment as the most prevalent form of intrinsic capacity decline among community-dwelling elderly individuals (Liu et al., 2024). This discrepancy could be attributed to variations in how the different dimensions of intrinsic capacity are weighted during assessments. Therefore, further large-scale studies are needed to develop standardized assessment tools tailored to the characteristics of elderly populations in China, which could provide a scientific basis for clinical practice in intrinsic capacity and contribute to the advancement of healthy aging.

### Predictive value of intrinsic capacity for falls in community-dwelling elderly

The findings of this study indicate that declines in the physical, psychological, and sensory dimensions were independently associated with an increased risk of falls among elderly individuals. The locomotive dimension, which reflects physical activity capacity, is particularly important as aging leads to the deterioration of various bodily functions, including decreased skeletal muscle strength, impaired coordination, and reduced central nervous system control, all of which contribute to a heightened fall risk (Cuevas-Trisan, 2019). Liu et al. (2021) conducted a 2-year longitudinal study involving 212 community-dwelling elderly individuals, finding significant associations between declines in the locomotive and psychological dimensions and fall risk, which is consistent with the results of this study. Additionally, studies by Long et al. (2022) indicated that elderly individuals with depression have a significantly higher fall risk, with a risk factor 9.03 times greater than that of non-depressed individuals. Sensory impairment is another critical factor, as reduced auditory input and limited visual-spatial awareness can further increase fall risk (Jiam et al., 2016). Research has also shown that cognitive impairment combined with hearing loss negatively impacts quality of life, leading to a decline in intrinsic capacity and an increased fall risk (Meng et al., 2023). Thus, when monitoring the intrinsic capacity of community-dwelling elderly individuals, it is essential to focus on the locomotive, psychological, and sensory dimensions. Early identification of declines in these areas, along with targeted interventions, can help effectively prevent falls.

### Predictive value of intrinsic capacity for disability in community-dwelling elderly

The results of this study also indicate that declines in the locomotive, cognitive, and psychological dimensions are independently associated with an increased risk of disability among elderly individuals. Research by Muneera et al. (2023) found that physical function, as an indicator of intrinsic capacity, is a significant predictor of difficulties in daily living activities. Additionally, depression and cognitive impairment were shown to be more predictive of disability than other dimensions of intrinsic capacity. Beard et al. (2019) demonstrated that intrinsic capacity is an independent risk factor for declines in both Activities of Daily Living (ADL) and Instrumental Activities of Daily Living (IADL) in elderly individuals and serves as a reliable predictor of care dependency. Zhao et al. (2021) found that elderly individuals with impairments in three or more intrinsic capacity domains had a tenfold increased risk of disability compared to those with no impairments. Therefore, declines in intrinsic capacity are a strong predictor of disability, underscoring the importance of monitoring intrinsic capacity and providing personalized interventions to delay the onset of disability among community-dwelling elderly individuals.

### Limitations

While this study holds significant theoretical and practical value, it also has several limitations. First, some of the follow-up assessments were conducted via phone calls, which may weaken the accuracy and authenticity of the data. Second, the study’s variable selection was limited, as it focused only on predicting falls and disability, failing to comprehensively capture other adverse outcomes related to the decline in intrinsic capacity. Additionally, the diagnosis of depression was based on screening tools and was not confirmed through clinical evaluation. Future research could address these limitations by including variables such as frailty and readmission rates to enhance the study’s design. Lastly, the study was limited to elderly individuals from the Hannan District of Wuhan, which restricts the ability to generalize the findings to elderly populations nationwide. To improve the scientific rigor and generalizability, future studies should expand the geographic scope and sample size to include community-dwelling elderly individuals from diverse regions across China.

## Conclusion

In conclusion, the decline in intrinsic capacity is relatively common among community-dwelling elderly individuals and serves as an independent risk factor for falls and disability within 1 year. Specifically, declines in locomotive, psychological, and sensory functions are associated with an increased risk of falls, while declines in locomotive, cognitive, and psychological functions are linked to a higher risk of disability. These findings suggest that the model of elderly care in communities should shift from a disease-centered approach to one that prioritizes functional ability. Early identification of intrinsic capacity decline in elderly individuals, coupled with targeted interventions based on specific levels of decline, can help reduce the occurrence of adverse health outcomes such as falls and disability, thereby fostering healthy aging in China.

## Data Availability

The raw data supporting the conclusions of this article will be made available by the authors, without undue reservation.
